# Response of basal metabolic rate to complete submergence of riparian species *Salix variegata* in the Three Gorges reservoir region

**DOI:** 10.1038/s41598-017-13467-0

**Published:** 2017-10-24

**Authors:** Shutong Lei, Bo Zeng, Shaojun Xu, Xiaoping Zhang

**Affiliations:** 1 College of Agriculture and Forestry Sciences, Linyi University, Linyi, 276005 China; 2 Key Laboratory of Eco-Environment in the Three Gorges Reservoir Region (Ministry of Education), School of Life Sciences, Southwest University, Chongqing, 400715 China; 3 Forestry College, Henan University of Science and Technology, Luoyang, 471003 China

## Abstract

One-year old seedlings of *Salix variegata* (submergence-tolerant) and *Cinnamomum camphora* (submergence-intolerant) were selected and subjected to complete submergence (2 m) for 1, 5, 10, and 20 days, to elucidate the submergence- tolerance mechanism of *S*. *variegata* in the Three Gorges reservoir region. The basal CO_2_ emission ratios (BCERs) and O_2_ consumption rates (OCRs) of leaf, stem, and root were determined. The basal O_2_ consumption rates (BOCRs) were calculated from the OCRs of different parts and their biomass allocations and used for evaluating the basal metabolic rate (BMR) of species with BCERs. The results showed that: (1) The BCERs of both species responded to flooding similarly, and no significant differences occurred between the submerged *S*. *variegata* (SS) and the submerged *C*. *camphora* (SC) seedlings, and between the control *S*. *variegata* (CS) and the control *C*. *camphora* (CC) seedlings. (2) The BOCRs of SS were significantly lower than those of SC on days 1 and 20, while no significant differences occurred between CS and CC for every duration. Therefore, the BMRs, evaluated from BOCRs rather than from BCERs, were related to submergence-tolerance of species, and the response of BMR to submergence would contribute to the survival of *S. variegata* seedlings under flooding.

## Introduction

Flooding is an abiotic stress with widespread effects on society and environment^1,2^. At present, flooding-environment interactions have gained extensive attentions from around the world^3–5^, of which response of plant species to flooding is primary^6,7^. Plants when subjected to flooding usually exhibit either escape strategy (keeping in close contact with the air via fast growth of seedlings)^8,9^ or quiescence strategy (maintaining survival in flooding without growth or even being dormant)^10^. The quiescence strategy has been proved to be an effective action for tolerating deep and prolonged flooding or frequently fluctuated flooding.

In the Three Gorges reservoir, water levels fluctuate regularly from 145 to 175 m in elevation, according to the water scheduling scheme of the Three Gorge reservoir^11^. Severe flooding with a maximum water level fluctuation of 30 m has caused several environmental problems, such as vegetation deterioration, soil erosion, and pollution^12^. At present, ecological restoration with well-adaptive plant species is considered an ideal optional action for mitigating environmental impacts of impounding water^13,14^. *Salix variegata* Franch., a riparian shrub species native to lowland ecosystems in the Three Gorges reservoir region, is a well-studied adaptive plant species for revegetation in the drawdown zone of the Three Gorges reservoir region. It can not only survive a severe complete submergence stress (2 m) for more than 180 days but also regrow quickly during desubmergence^15^. *S*. *variegata*, when subjected to complete submergence, presents several responses for survival, such as being out of growth^16^, reducing carbohydrate consumption, postponing flowering, and adjusting reproductive allocations^12^. These performances indicate that *S*. *variegata* is a species following quiescence strategy for tolerating flooding and would downregulate energy-dependent physiological activities for survival^17^, which isrelated to the plant basal metabolism. However, whether the response of basal metabolism to flooding contributed to its tolerance or not is still unclear even after 10 years of research on the submergence mechanisms of *S*. *variegata*.

Basal metabolism is the minimum level of energy required to sustain the vital functions of organisms and has been frequently used in investigating the adaptive capacities of organisms to external environments^18–20^. However, many studies of basal metabolism were conducted for animals^21^, and few studies have paid attentions to the response of plant basal metabolism to complete submergence. The fact that plants following the quiescence strategy can tolerate long duration or deep flooding^22–24^ is probably attributed to the low basal metabolic level. The basal metabolic level of organisms is measured using the basal metabolic rate (BMR), which is calculated from O_2_ consumption per unit time under given conditions^25^. It is convincible to evaluate the energy-dependent metabolism level by investigating the changes of O_2_ (aerobic respiration substrates) for animals. Evaluating BMR of plants is more complex than that of animals. On the one hand, plants could produce O_2_ via photosynthesis besides consumption, which might be affected by stress^26–28^. On the other hand, plants would conduct anaerobic metabolism under stress, especially for flooding stress. Therefore, the effect of growth and anaerobic metabolisms must be taken into consideration when evaluating the response of plant BMR to flooding.

Generally, capacities of conducting underwater photosynthesis and upregulating anaerobic metabolic level are two of the key factors of submergence-tolerant plants^7,29^. Plants obtain energy for physiological activities from aerobic and anaerobic metabolism simultaneously under flooding, greatly increasing the difficulty of measuring the BMR. However, investigating the BMR level of plants is still feasible under controlled conditions. First, the essence of BMR is the energy metabolic level, which is dependent on the hereditary feature^30^. As the main pathway of producing energy, respiratory metabolism of plants is related to its BMR^31^. Next, the respiratory metabolic level of plants can be measured by changes in substrate^32^ or enzymes activities^33^.

At present, the O_2_ consumption rate (OCR) and CO_2_ emission ratio (CER) are frequently used for investigating aerobic respiration^34^. The enzyme activities of fermentation, such as pyruvate decarboxylase (PDC), alcohol dehydrogenase (ADH), lactic dehydrogenase (LDH), and so on, are often used to evaluate anaerobic respiration^34^. OCR and CER can reflect the overall metabolic level of aerobic and anaerobic respiration under flooding stress. O_2_ is consumed in anaerobic respiration indirectly, maintaining redox equilibrium of plants as an efficient oxidant^35^. CO_2_ is released via the ethanol fermentation, the main pathway of the anaerobic respiration of plants^36^. However, no effective system is available for measuring the overall respiration of plants under flooding at present, and few trials have been conducted to elucidate plant BMR from respiration.

In the present study, the OCR and CER of two plant species were measured to investigate the interactions of BMR and submergence in a well-designed and carefully controlled experiment. First, the experiment was conducted in the winter season, which not only reduced the growth impacts but also mimicked winter flooding in the drawdown zone of the Three Gorges reservoir. Next, *S*. *variegata* and the control species were C3 plant species. Besides different submergence-tolerance abilities, the respiratory quotient of C3 plants was 1^36^, and thus the OCR and CER could be interconverted equally. It was the key factor that determined the success probability of the experiment. Finally, seedlings were placed in a dark room for 24 h, before measurement, to consume the photosynthetic products produced on the day. The OCR and CER of total seedlings were defined as basal OCR (BOCR) and basal CER (BCER), respectively.

One-year old seedlings of *S*. *variegata* (submergence-tolerant species) and *Cinnamomum camphora* (intolerant species) were selected and complete submergence was conducted (2 m) for 1, 5, 10, and 20 days in the present study. The BCERs and OCRs of the leaves, stems, and roots of both species were determined, and the BOCRs were investigated as the average OCR of seedlings. The relationship between submergence tolerance of plants and their BMRs was elucidated from BCERs and BOCRs, and the index was also selected for evaluating tolerant abilities.

## Results

### Biomass remained stable under submergence

**Figure 1:**
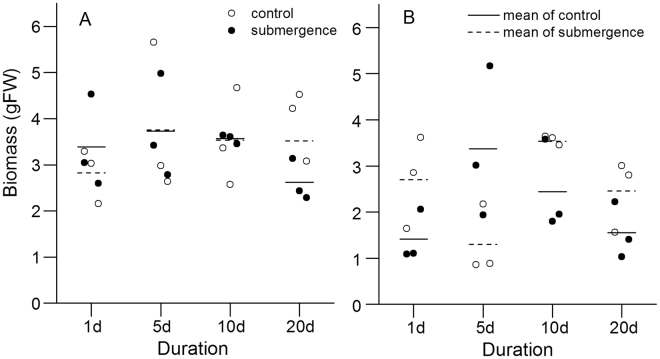
Biomass of *S*. *variegata* and *C*. *camphora*. (**A**) *S*. *variegata* and (**B**) *C*. *camphora*. The hollow circle indicates biomass of the control seedlings; the solid circle indicates biomass of the submerged seedlings; the dashed line indicates mean biomass of control; and the full line indicates mean biomass of the submerged seedlings.

The biomass of *S*. *variegata* ranged from 2.154 to 5.653 g (CS) and 2.284 to 4.980 g (SS), with the means being 3.515 ± 0.298 (±SE) g (CS) and 3.389 ± 0.584 g (SS), respectively (Fig. 1A). The biomass of *C*. *camphora* ranged from 0.854 to 3.640 g (CC) and 1.027 to 5.162 g (SC), with the means being 2.507 ± 0.305 g and 2.193 ± 0.349 g, respectively (Fig. 1B).

No significant differences were found in the biomass of CS, SS, and SC with different duration (ANOVA, CS, *P* = 0.637; SS, *P* = 0.064; and SC, *P* = 0.196), except in the biomass of CC (ANOVA, *P* = 0.034) (Fig. 1). The Duncan’s multiple-range test results showed that the difference of CC occurred on days 5 and 10, with the former being significantly lower than the latter.

The *t* test results showed no significant differences in the biomass of the two species between treatment and control, or between species for every duration, except for that of SS and SC on day 1 (*t* test, *P* = 0.041).

### Biomass allocation responded slightly to submergence

**Figure 2:**
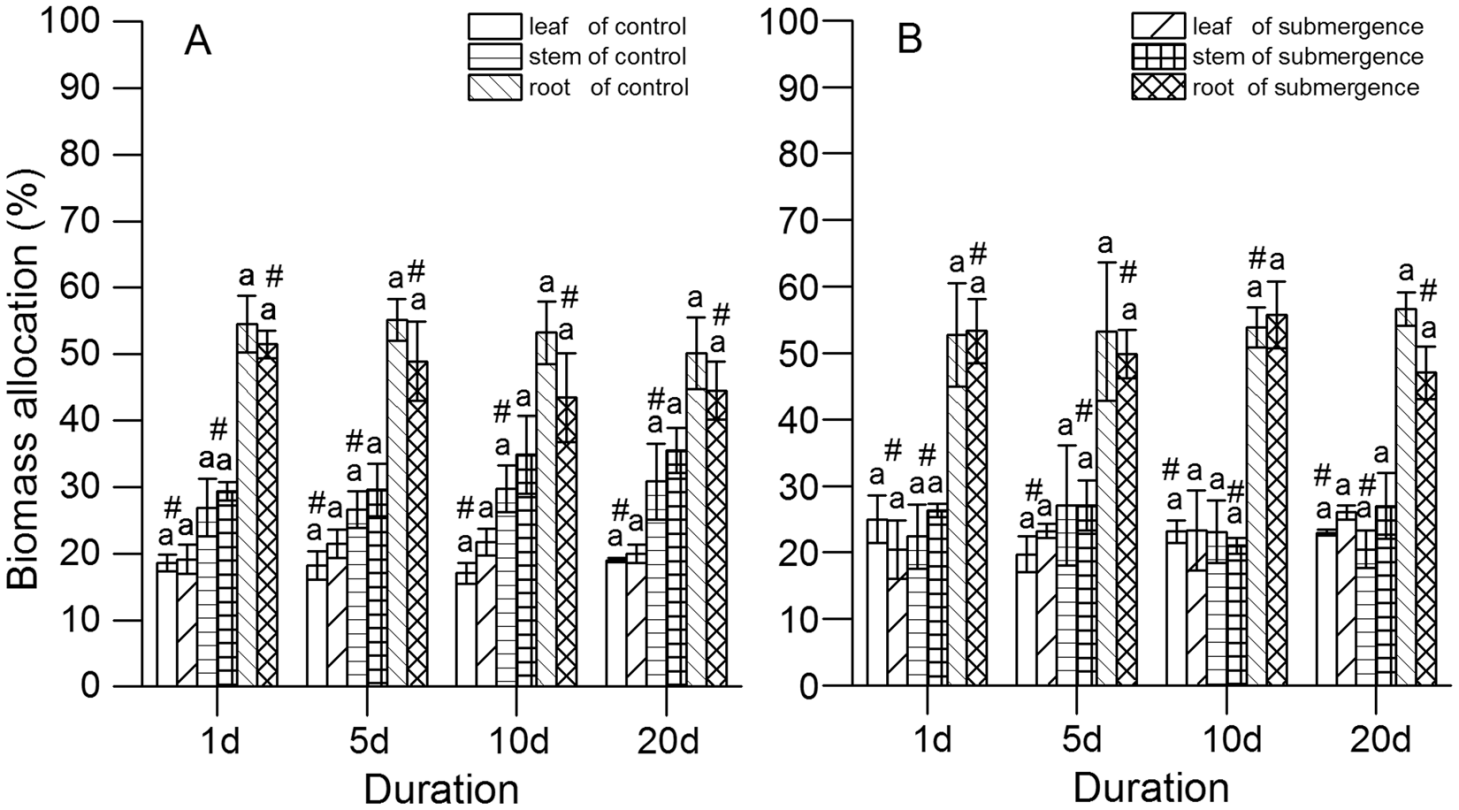
Biomass allocations of *S*. *variegata* and *C*. *camphora*. (**A**) *S*. *variegata* and (**B**) *C*. *camphora*. Values are mean ± standard error of mean < SE has already been defined in text > based on three independent assays from ANOVA. The letter “a” indicates no statistical significance among duration (*P* > 0.05) according to the ANOVA. ^#^Indicates no difference between the submerged seedlings and the control according to the *t* test (*P* > 0.05).

The biomass allocation ranked as root > stem ≥ leaf in both species, with root being about a half of the biomass (Fig. 2).

No significant differences were found in biomass allocations of leaf, stem, and root for the two species (ANOVA, leaf of CS, *P* = 0.802; stem of CS, *P* = 0.862; root of CS, *P* = 0.856; leaf of SS, *P* = 0.761; stem of SS, *P* = 0.584; root of SS, *P* = 0.662; leaf of CC, *P* = 0.517; stem of CC, *P* = 0.878; root of CC, *P* = 0.976; leaf of SC, *P* = 0.781; stem of SC, *P* = 0.523; and root of SC, *P* = 0.555).

The *t* test results showed that no significant difference occurred in the biomass allocation of the two species between treatment and control, or between species for all parts and duration, except that of leaf between species on day 20 (*t* test, control, *P* = 0.002; submerged, *P* = 0.027).

### BCER of *S*. *variegata* and *C*. *camphora* responded to submergence in a similar manner

**Figure 3:**
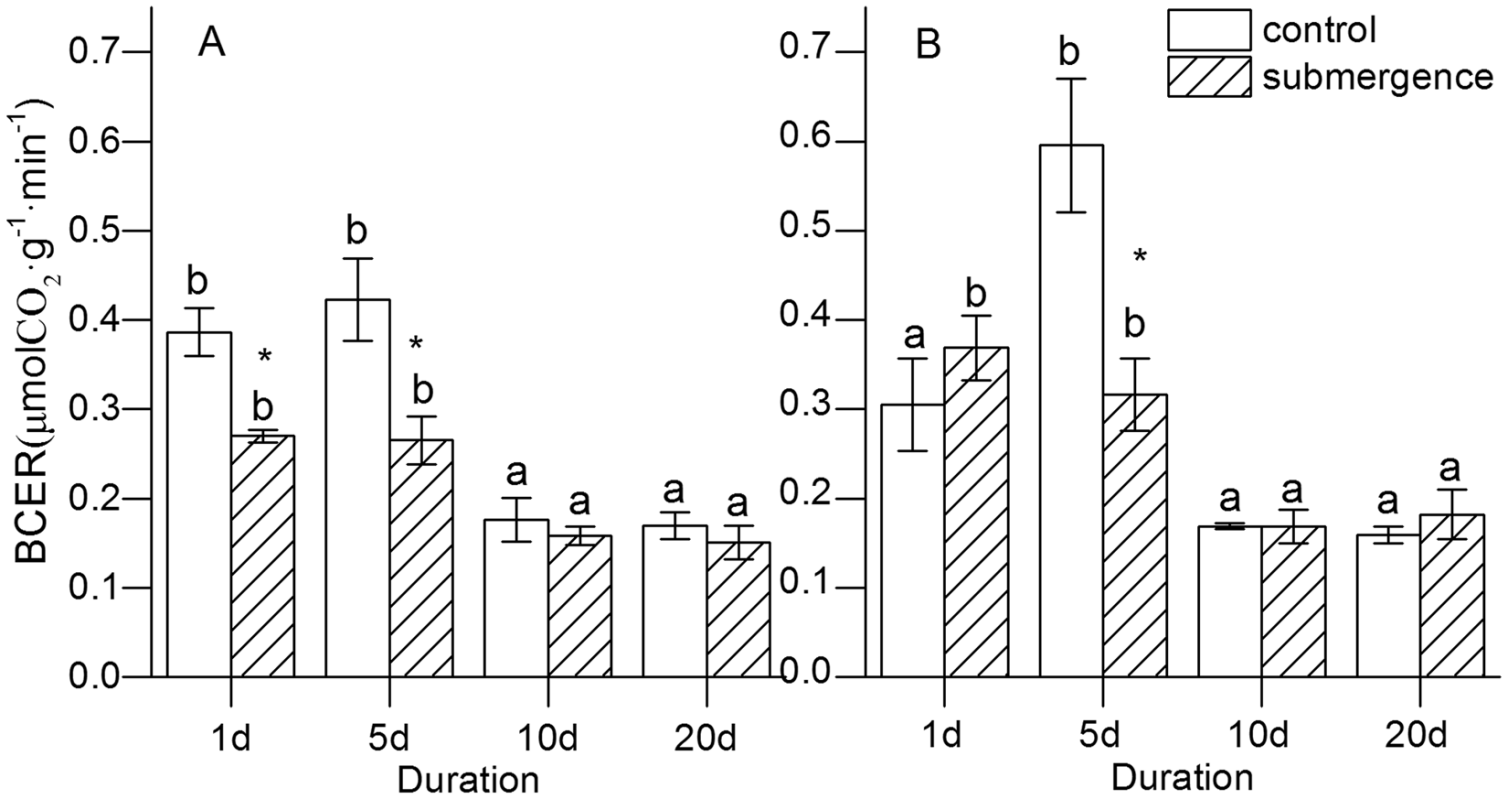
Response of BCERs to submergence for *S*. *variegata* and *C*. *camphora*. (**A**) *S*. *variegata* and (**B**) *C*. *camphora*. Values are mean ± standard error of mean <SE has already beendefined in text> based on three independent assays from ANOVA. Different letters indicate statistical significance (*P* < 0.05) according to the Duncan’s test. ^*^Indicates difference between the submerged seedlings and the control according to the *t* test (*P* < 0.05).

The BCERs of SS and SC responded to submergence significantly, with differences among various duration (ANOVA, SS, *P* = 0.002; SC, *P* = 0.005). The BCERs of SS and SC on days 10 and 20 were significantly lower than those on days 1 and 5 (Fig. 3). The *t* test results showed significant differences between treatment and control on days 1 and 5 for *S*. *variegata*, and on day 5 for *C*. *camphora*, while no significant differences occurred between species for every duration.

### OCR of *S*. *variegata* and *C*. *camphora* responded differently to submergence

**Figure 4:**
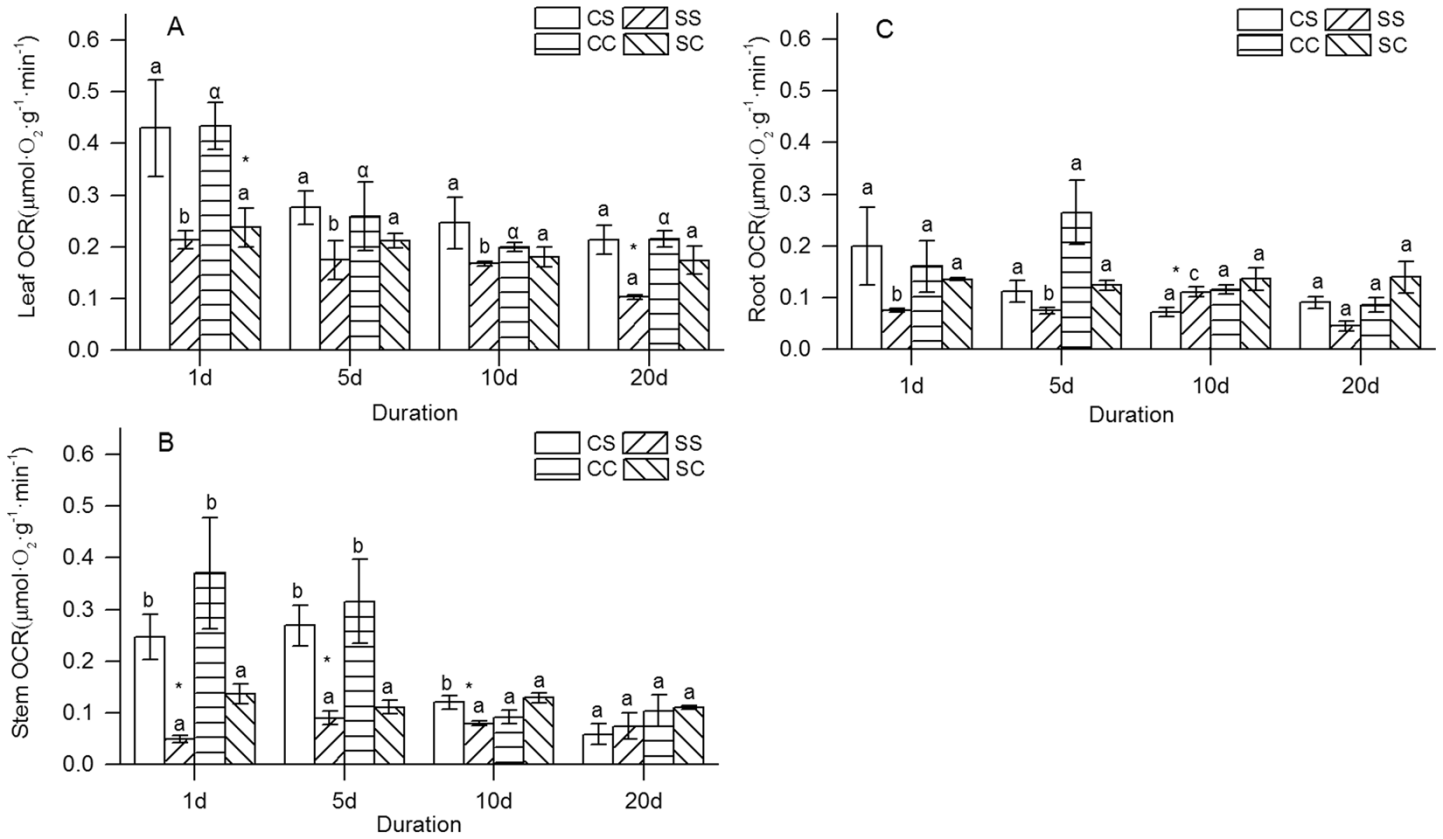
Response of the OCRs to submergence for *S*. *variegata* and *C*. *camphora*. (**A**) OCRs of leaf, (**B**) OCRs of stem, and (**C**) OCRs of root. Values are mean ± standard error of mean < SE has already been defined in text > based on three independent assays from ANOVA. Different letters indicate statistical significance (*P* < 0.05) according to the Duncan’s test (homogeneity of variances was assumed). ^*^Indicates difference between the submerged seedlings and the control according to the *t* test (*P* < 0.05). The Greek letter α in A indicates no difference between the control seeding according to the Tamhane’s test (homogeneity of variances was not assumed). The abbreviations, CS, SS, CC and CS, indicate the control seedlings of *S*. *variegata*, the submerged seedlings of *S*. *variegata*, the control seedlings of *C*. *camphora*, and the submerged seedlings of *C*. *camphora*, respectively.

The OCRs of leaves of *S*. *variegata* and *C*. *camphora* responded to submergence in a similar manner, with the treatment being lower than the control on days 20 and 1, respectively (*t* test, *S*. *variegata, P* = 0.017; *C*. *camphora*, *P* = 0.029, Fig. 4A). The *t* test results also showed no significant differences between species for every duration. A significant difference was observed in the OCRs of leaves among various duration for SS (ANOVA, *P* = 0.013), with those on day 20 being significantly lower than those on days 1, 5, and 10 (Fig. 4A) (Duncan’s multiple-range test), while no significant difference occurred for SC (ANOVA, *P* = 0.342; Fig. 4A).

The response of OCRs of stems of *S*. *variegata* differed compared with that of *C*. *camphora*. A difference between treatment and control for *S*. *variegata* was observed on days 1, 5, and 10, while no difference occurred for *C*. *camphora* (Fig. 4B). The *t* test results showed a significant difference in the OCRs of stems of SS and SC on days 1 and 10, while no significant difference was found in that of CS and CC for every duration. No significant differences were observed in the OCRs of stems of SS and SC during duration (ANOVA, SS, *P* = 0.311; SC, *P* = 0.435), while it decreased with duration for control (ANOVA, CS, *P* = 0.005; CC, *P* = 0.023) (Fig. 4B).

A significant difference was observed in the OCRs of roots of SS among various duration (ANOVA, *P* = 0.002), with it being higher or lower than CS on days 10 and 20, respectively. However, no significant difference occurred in the OCRs of roots of the *C*. *camphora* seedlings among duration (ANOVA, *P* = 0.941), or between treatment and control for every duration. The *t* test results showed a significant difference in the OCRs of roots of SS and SC on days 1, 5, and 20, while no significant difference was found in the OCRs of roots of CS and CC for every duration (Fig. 4C).

### BOCR of *S*. *variegata* and *C*. *camphora* responded differently to submergence

The BOCR was evaluated as an average OCR of seedlings, which was calculated using the following formula: BOCR = Σ (OCR × biomass allocation).

**Figure 5:**
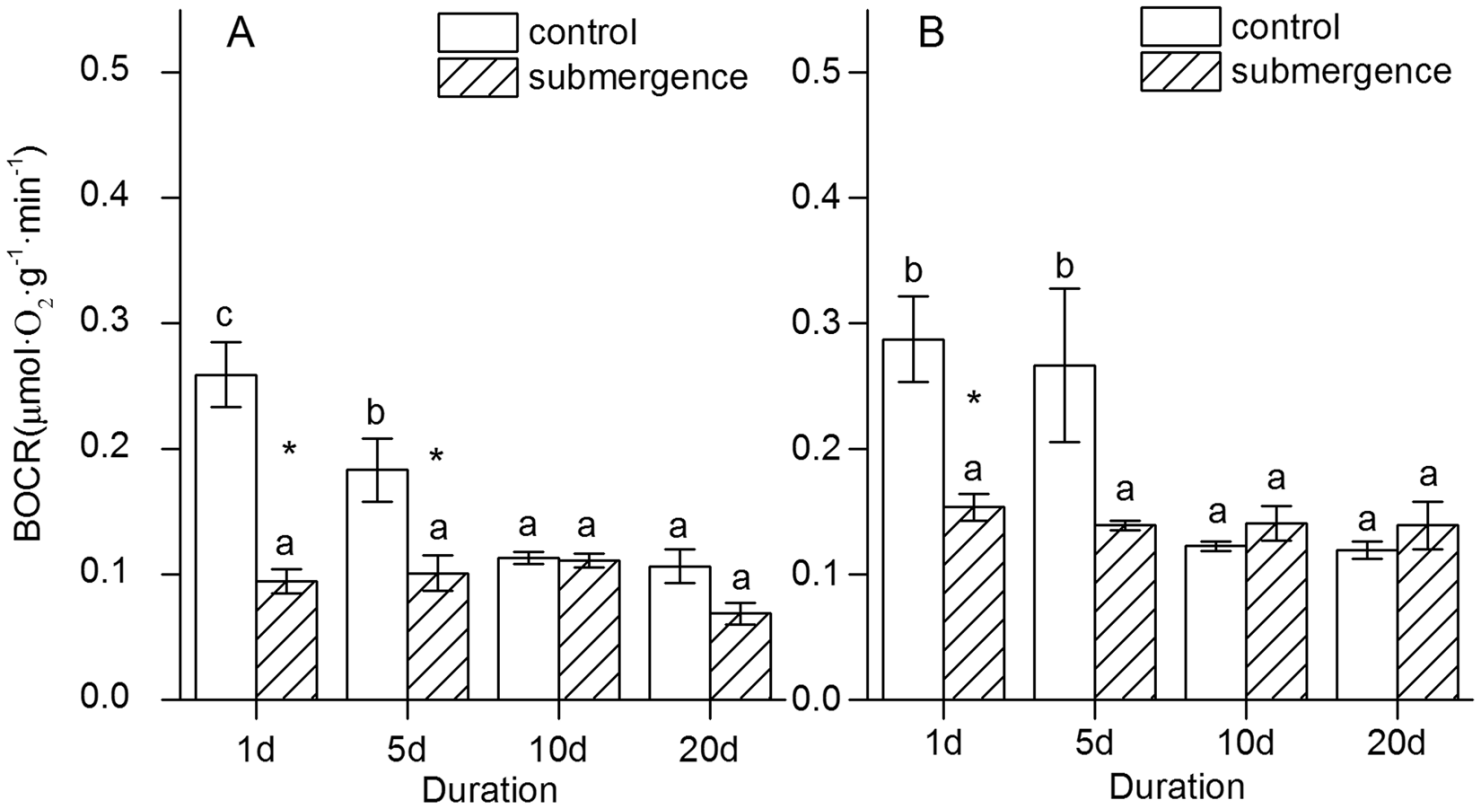
Effect of flooding on the BOCRs of *S*. *variegata* and *C*. *camphora*. (**A**) *S*. *variegata* and (**B**) *C*. *camphora*. Values are mean ± standard error of mean < SE has already been defined in text > based on three independent assays from ANOVA. The BMR was evaluated from the average OCR of seedlings, which was calculated using the following formula: BOCR = Σ (OCR × biomass allocation). Different letters indicate statistical significance (*P* < 0.05) according to the Duncan’s test. ^*^Indicates difference between the submerged seedlings and the control, according to the *t* test (*P* < 0.05).

No significant difference was found in the BOCR of SS (ANOVA, *P* = 0.084), while a difference was found in that of CS (ANOVA, *P* = 0.002). The BOCR of the CS seedlings decreased significantly, with the BOCR on days 10 and 20 being significantly lower than that on days 1 and 5 (Fig. 5A). The *t* test results showed that the BOCR of the SS seedlings was significantly lower than that of the control on days 1 and 5.

No significant difference was found in the BOCR of the SC seedlings (ANOVA, *P* = 0.828), while a difference was found in that of the CC seedlings (ANOVA, *P* = 0.015). The BOCR of the CC seedlings decreased significantly, with the BOCRs on days 10 and 20 being significantly lower than those on days 1 and 5 (Fig. 5B). The *t* test results showed a significant difference in the BOCRs of the SC and CC seedlings only on day 1.

The *t* test results showed a significant difference in the BOCRs of SS and SC on days 1 and 20, while no significant difference was found in the BOCRs of CS and CC for every duration (Fig. 5).

### OCR and BCER of *S*. *variegata* were less affected by biomass and temperature compared with those of *C*. *camphora*

Generally, mechanism levels of plants are related to their developed status and habitat conditions^36^. To elucidate correlation of the BMR of *S*. *variegata* and flooding, effects of developed status and habitat on plant BMR must be evaluated and eliminated. In the present study, effects of biomass (developed status) and temperature (habitat factor) on BMR were investigated via correlation analysis, in which the temperatures were those recorded by apparatus directly during measuring.

No significant correlations were found between OCRs and biomass for both *S*. *variegata* and *C*. *camphora* (Table 1). No significant correlations occurred between BCERs and biomass for *S*. *variegata*, while significant correlation was observed between BCER and biomass for *C*. *camphora*.

**Table 1:** Pearson’s correlations of OCRs and BCERs to biomass or temperature.

Species	Index		Biomass	Temperature
*S*. *variegata*	leaf OCR	CS	−0.252	0.479
		SS	0.168	0.306
	stem OCR	CS	−0.548	0.894**
		SS	0.085	−0.022
	root OCR	CS	−0.074	0.453
		SS	0.061	0.425
	BCER	CS	−0.434	0.296
		SS	0.221	−0.271
*C*. *camphora*	leaf OCR	CC	−0.091	0.591*
		SC	−0.363	0.026
	stem OCR	CC	−0.47	0.770**
		SC	−0.039	−0.036
	root OCR	CC	−0.488	0.703**
		SC	−0.254	−0.366
	BCER	CC	−0.811**	0.537
		SC	−0.265	−0.462

Biomass in the table indicates the total fresh weight of the total seedling; and temperature indicates the temperature on measuring the certain index. Data in the table indicate the Pearson’s correlation values (*r* ^2^). ^*^Indicates correlations occurred between OCR and BCER to biomass or temperature according to the Pearson’s correlation test (*P* < 0.05). ^**^Indicates correlations occurred between OCR and BCER to biomass or temperature, according to Pearson’s correlation test (*P* < 0.01). The abbreviations, CS, SS, CC and CS, indicate the control seedlings of *S*. *variegata*, the submerged seedlings of *S*. *variegata*, the control seedlings of *C*. *camphora*, and the submerged seedlings of *C*. *camphora*, respectively.

No significant correlations were observed between OCRs and temperature, and BCERs and temperature for SS and SC. Significant correlations were observed between OCRs and temperature for the leaf, stem, and root of CC, and the stem of CS.

## Discussion

Generally, CER and OCR are the two key indices for investigating energy-dependent metabolisms of plants, with the former being more widely applied than the latter^34^. The BCER and BOCR were investigated directly or indirectly to elucidate the relationship between submergence tolerance of plants and their BMR in the present study. The BCER must be a preferred index, because the determination of BCER was nondestructive and easy. Moreover, the BCERs of both treatment and control were determined by the same measuring system, which could reduce the systematic error caused by different instruments. However, the BCERs of both species responded to flooding in a similar manner, and no significant differences were observed between CS and CC, and SS and SC (Fig. 3). This indicated no significant interrelation between BCER and submergence tolerance. It was probably due to the interference of biomass. A highly significant negative correlation (Tab. 1) was found in the BCER and the biomass for CC in the present study, indicating that the BCER of seedling would be affected by maturity. Nevertheless, the BCERs of the two species were the average CER of total seedling, not considering the allocation of young and mature part. Therefore, the BCER determined in the present study was inappropriate to be used for evaluating the BMR of plants.

The OCRs of leaves of SS and SC were significantly lower compared with those of control on days 20 and 1, respectively (Fig. 4A), indicating that the tolerant and intolerant species would both reduce O_2_ consumption under water but with a bit of difference. The difference probably resulted from different abilities of conducting underwater photosynthesis. A previous study reported that the *S*. *variegata* seedlings could conduct photosynthesis on complete submergence, while the *C*. *camphora* seedlings could not^39^. No significant difference was found in the OCRs of leaves between species in the present study, irrespective of treatment and control (Fig. 4A). It indicated that the OCR of leaf was of only a little relation to submergence tolerance. Parad *et al*. (2016)^40^ reported that when subjected to continuous flooding, leaves of *Quercus castaneifolia* experienced precocious senescence and wilting. Therefore, the OCR of leaf was not an ideal index for evaluating submergence tolerance of plants.

The OCRs of stems of SS were significantly lower than those of CS on days 1, 5, and 10 of submergence, while no significant difference occurred between SC and CC for every duration (Fig. 4B). This indicated that the OCR of stem of *S*. *variegata* responded to flooding more strongly than that of *C*. *camphora*. Generally, a stem conducts transportation of nutrition and gas from the leaf to the root, and is affected by the O_2_ yield of leaves. The decrease in the OCR of stem for the SS seedlings was probably due to its initial response for the decrease in the available O_2_ under submergence stress. Previous studies reported that the *S*. *variegata* seedlings would reduce photosynthesis^39^ and also conduct defoliation under flooding^41^, which would decrease O_2_ production. The OCRs of stems of SS were significantly lower compared with those of SC on days 1 and 10 in the present study, while no differences occurred between the OCRs of stems of CS and CC for every duration (Fig. 4B). It further demonstrated that *S*. *variegata* consumed less O_2_ for survival compared with *C*. *camphora*, which has a great significance for tolerating long-duration flooding. Therefore, the OCR of stem is an optional index for estimating submergence tolerance of plants, although it is sensitive to temperature (Tab. 1).

The OCRs of roots of SS increased first, followed by a decrease with duration (Fig. 4C), indicating that O_2_ consumption of roots was complex. It is because O_2_ of roots comes from multiple channels and is affected by relevant elements. On the one hand, roots mainly obtain O_2_ from the photosynthesis of leaves, and are affected by the photosynthetic level of leaves and the transfer efficiency of O_2_ of stems. On the other hand, roots could absorb O_2_ directly from the external environment via special roots, such as aerial roots and water roots, which is affected by the O_2_ level in the environment and root structure. A previous study reported that *S*. *variegata* could take water roots^42^, while *C*. *camphora* could not^16^. Parad *et al*. (2013)^43^ reported that the flooded plants of *Pyrus boissieriana* developed adventitious roots, while the non-flooded ones did not. Therefore, the OCR of root is related to the submergence tolerance of plants, but it is not an available index for evaluating submergence tolerance.

In the present study, the BOCR was calculated from OCRs (leaf, stem, and root) and its biomass allocations. It seemed that the BOCR would be affected by the possible differences in OCRs and biomass allocations between species. Luckily, the both selected species in the present study, *S*. *variegata* and *C*. *camphora*, had several similar traits, ensuring that the BOCRs were compared among species directly. First, no significant differences were found in biomass (except for the biomass of CC on day 5) (Fig. 1) and biomass allocations (except for the leaf percentage on day 20) of the two species for every duration (Fig. 2), including the submerged and the control seedlings. Next, no significant differences occurred in the OCRs of leaves, stems, and roots for CS and CC (Fig. 4). Therefore, the BOCRs could reflect the integral level of O_2_ consumption of plants, and interrelations of BMR and submergence tolerance. Moreover, the BOCRs of SS and SC seedlings could also be compared with each other directly, which could not only eliminate the effects of systematic errors of different measuring instruments but also reduce the effects of temperature on the OCRs of plants.

The BOCR of the SS seedlings was significantly lower compared with that of SC in the present study, while no significant difference was found in the control seedlings of the two species (Fig. 5). This indicated that the tolerant plants might need less energy for maintaining physiological activities than the intolerant plants. Lei *et al*. (2012)^16^ reported that both *S*. *variegata* and *C*. *camphora* could upregulate the activities of antioxidant enzymes, such as superoxide dismutase and peroxidase. However, the overall peroxidase level of *S*. *variegata* was significantly higher compared with that of *C*. *camphora* on day 15 of submergence, although the former increased less than the latter. Ye and Zeng (2013)^41^ also found that nonstructural carbohydrate contents of *S*. *variegata* reduced insignificantly when subjected to complete submergence (2 m) for 150 days. Therefore, the BOCR level of plants was closely correlated with its tolerance ability and might be a good index for selecting adaptive species of revegetation in drawdown zones.

OCRs of SS and SC remained relative stable in the present study, about 0.1–0.2 (μmol·O_2_·g^−1^·min^−1^) (Fig. 4). It indicated that both *S*. *variegata* and *C*. *camphora* would consume O_2_ persistently upon submergence, requiring the plants to obtain O_2_ from water or preserve abundant O_2_ in seedlings. Previous studies extensively explored the mechanisms of obtaining O_2_ under flooding to cope with hypoxia stress^44–46^. Adventitious roots would be developed for absorbing O_2_ from water^47^, and aerenchyma is usually formed for diffusing gas among organs^48,49^. Dissolved oxygen is considered to play a vital role in the submergence tolerance of plants^50^. Generally, the *Salix* species, as riparian plants, would depend on aerenchyma for dealing with hypoxia stress on a wetland habitat^51,52^, while few morphology responses to flooding have been reported for *Cinnamomum* species. Therefore, it is possible the different responses to hypoxia stress, including morphological and physiological adaptation, that resulted in different submergence tolerances of plants.

The relationship between OCRs of stem and submergence tolerance of species, rather than the OCRs of leaf and root, was an unexpected finding. In previous studies, the focus of submergence tolerance mechanisms for plants was on responses of leaves and roots to flooding ^7, 17^; few studies have been conducted on stem physiology of plants during flooding in *S*. *variegata*. The OCR of stem of SS was lower compared with that of CS on day 1 of submergence in the present study, while no significant difference occurred for OCRs of leaf and root (Fig. 4). The quick response of the OCR of stem to flooding indicated that the transport efficiency of *S*. *variegata* for nutrition and gas might be affected by flooding significantly, which would probably influence its tolerant abilities. Therefore, the stem physiology of *S*. *variegata* must be referred in further studies to conduct revegetation with the species in the drawdown zone of the Three Gorges reservoir region.

The present study had certain limitations. A few factors were not taken into consideration, and some results, such as the fluctuation root OCR under flooding, could not be explained clearly. However, submergence tolerance of plants is known to be related to their basal metabolism. The OCRs of stem and the BOCRs can be used as good alternative indices for evaluating submergence tolerance of plants. Finally, this study was indeed a successful attempt for investigating the tolerance mechanisms of plants under flooding, although many details still need to be explored further.

## Materials and Methods

### Plant materials

One-year old seedlings of *S*. *variegata* and *C*. *camphora* were collected in June 2008. *S*. *variegata* seedlings were taken from a riparian shrub community located on the banks of the Jialing River, and *C*. *camphora* seedlings were collected from the campus of Southwest University, Chongqing, China. The seedlings of both species were of similar height, ranging from 15 to 20 cm. They were transplanted into plastic containers (18 × 15 cm^2^ high) with 3 kg soil (loam, collected from an experimental garden of the Southwest University). Sixty seedlings for each species were cultivated in the present study, supplying enough materials for treatments (3–5 replicates for each treatment) and pretreatments. No endangered or protected species is involved in the present study.

**Figure 6:**
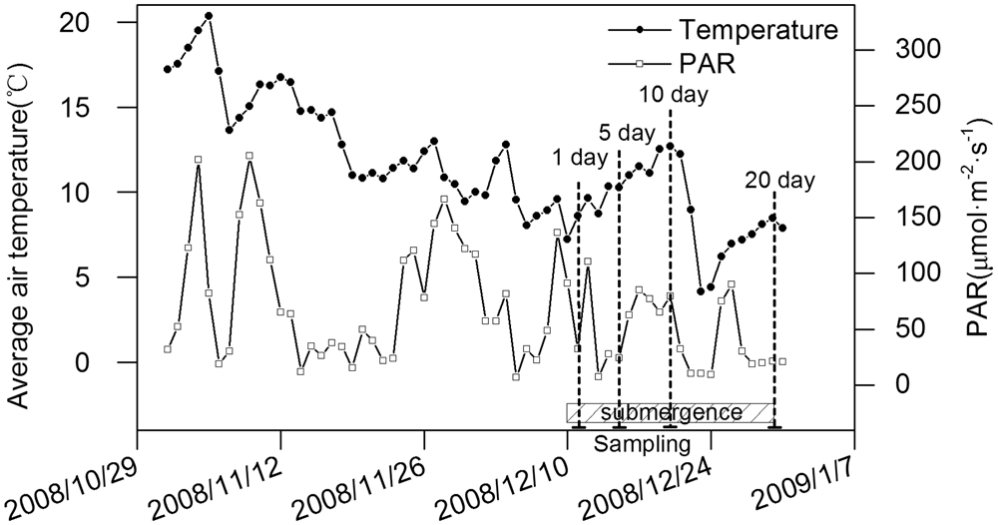
Changes in average air temperature and PAR. Shallow with inclined lines indicate submergence duration, and the dash lines show the date for sampling at 1, 5, 10, and 20 days of treatment.

The growth conditions were recorded using the DynaMet Weather Station (Dynamax Inc., MI, USA). The average air temperature and photosynthetically active radiation (PAR) are shown in Fig. 6, and the experimental procedures are shown in Fig. 6.

### Flooding treatment and sampling

At the beginning of December 2008, the seedlings were subjected to flooding treatments, which consisted of a set of submersion treatments and control (nonsubmersion). The treatment groups were named as follows: the control seedlings of *S*. *variegata* (CS), the submerged seedlings of *S*. *variegata* (SS), the control seedlings of *C*. *camphora* (CC), and the submerged seedlings of *C*. *camphora* (SC). The submersion treatment was conducted in a concrete tank (2-m long × 2-m wide × 2.2-m high) for four duration (1, 5, 10, and 20 days) with a water level of 2 m above the soil surface. The nonsubmerged seedlings were watered daily to maintain the field capacity and allowed to drain freely. Seedlings were collected randomly (*n* = 3) at the end of each treatment period, and one seedling was a replicate.

All the collected submerged seedlings were washed carefully under water without air contact, while the nonsubmerged seedlings were washed in the air. Then, the seedlings were placed in a dark room for 24 h to meet the requirements of determining the basal metabolism. Both species showed no growth in winter and mainly survived based on daily photosynthesis and previous reserves. Therefore, temporary cultivation for 24 h in the dark was considered to eliminate the effects of growth respiration. Soil lixivium (1 kg soil was put into 10 L of water for 48 h) was used to maintain fundamental physiological activities of the seedlings. The seedlings of the treatment group were kept completely submerged by lixivium, while the control seedlings were only subjected to waterlogging for roots.

### Measurement of BCERs and OCRs

The measurement of BCERs and OCRs of the two species were conducted in a dark room. A lamp (5 W, green light) was used to supply light for operating apparatus. The BCERs of the two species were determined using a Li-6400 Portable Photosynthesis System (Li-COR Inc., NE, USA), monitoring changes in CO_2_ (μmol·CO_2_· g^−1^·min^−1^) (converted from CO_2_ flux) via a 6400–09 Soil Chamber (Li-COR Inc., NE, USA). The whole procedure of measuring BCERs must be accomplished within 15 min, avoiding the effects of postsubmergence. A previous study reported that the physiological activities of plants were stable in desubmergence for 30 min^37^. Therefore, the limit of 15 min for operating was considered to meet the requirement of reducing the interference of desubmergence.

The OCRs of the leaf, stem, and root of the control groups were determined using the 6400–01 Standard Chamber, monitoring changes in CO_2_ (μmol·CO_2_·g^−1^·min^−1^). The OCRs of the submerged seedlings were analyzed using a Chloro-Lab2 liquid-phase O_2_ measurement system (Hansatech, Poole, UK), measuring changes in O_2_ (μmol·O_2_·g^−1^·min^−1^). The submerged seedlings were kept in water during sample preparation and determination. Albolene was used to guard against oxidation of the control seedlings.

### Statistical analyses

Data transformation was hardly inevitable to compare differences of OCRs between the treatment and control groups. As the respiratory quotient of C3 plants was 1^38^, CER in the leaf, stem, and root of the control group (μmol·CO_2_·g^−1^·min^−1^) was transformed to be consistent with the OCR in the submerged group (μmol·O_2_·g^−1^·min^−1^). The BOCR was evaluated as an average OCR of seedlings, which was calculated using the following formula: BOCR = Σ (OCR × biomass allocation).

Statistical analyses were performed using the Statistical Product and Service Solutions version 19.0 (SPSS Inc., IL, USA). A one-way analysis of variance (ANOVA) was used to compare the means of BCER, OCR, and BOCR in the submerged seedlings and control. Before conducting ANOVA, the homogeneity of variances must be tested. If homogeneity of variances was assumed, the Duncan’s multiple-range test was used to test whether the BCER, OCR, and BOCR differed between duration. If the homogeneity was not assumed, the Tamhane’s test was used. The independent samples *t* test (*t* test) was used to test differences between the submerged seedlings and the control, and between species for each treatment. All the results were presented as mean values [±standard error (SE)] obtained from three independent replicates.

**Figure 7:**
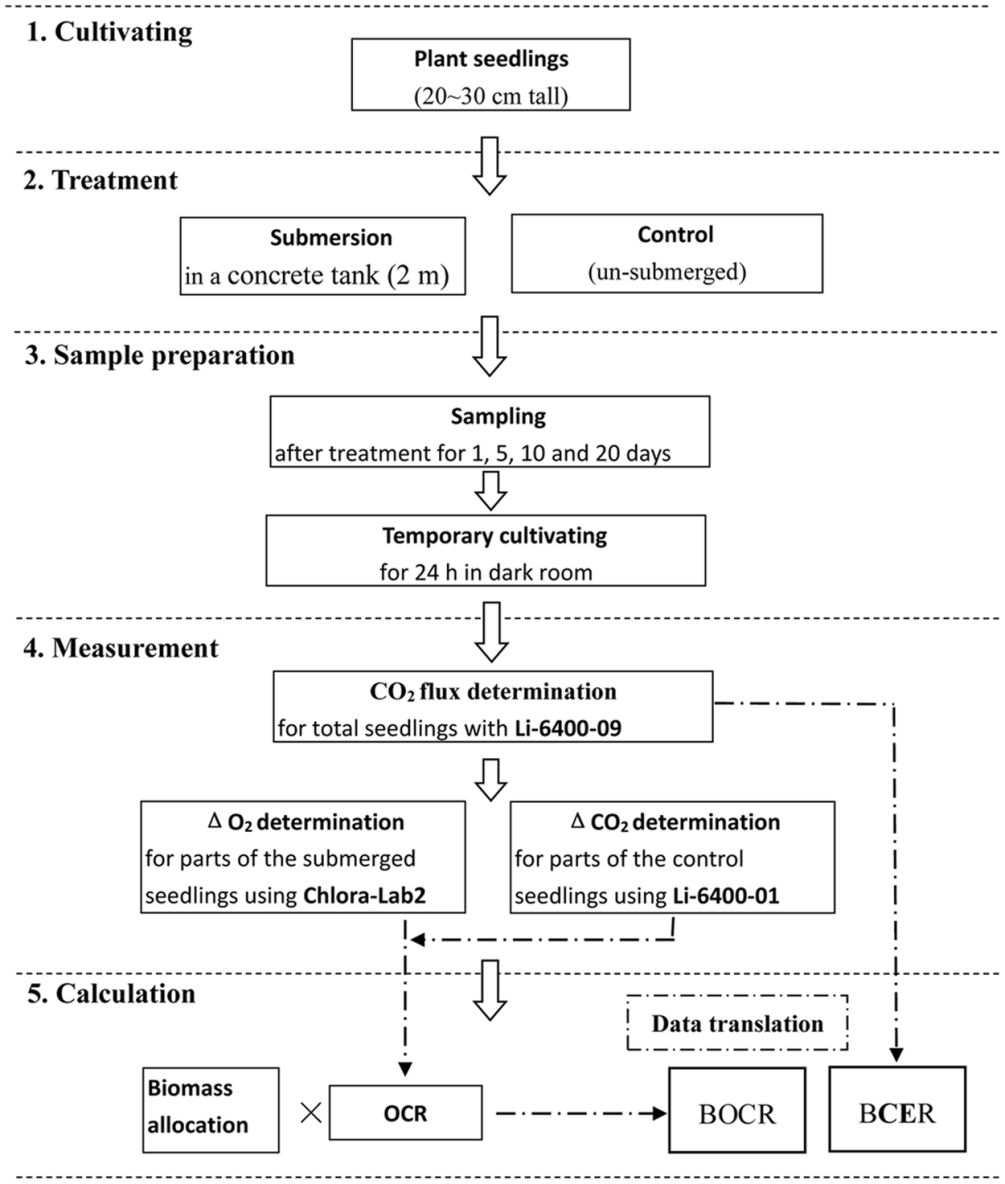
Response of BCERs and BOCRs to complete submergence for *S*. *variegata* and *C*. *camphora*. FW, fresh weight; Chloro-Lab2, liquid-phase O_2_ measurement system; Li-6400-09, portable photosynthesis system with soil chamber; Li-6400-01, portable photosynthesis system with the standard chamber.

Figures 1–6 were drawn using Origin 9.0 (OriginLab, MA, USA), while Figure 7 was drawn using Microsoft Office 2016.

### Data Availability

All data generated or analysed during this study are included in this published article.

## References

[1] Grames J, Prskawetz A, Grass D, Viglione A, Blöschl G (2016). Modeling the interaction between flooding events and economic growth. Ecol Econ..

[2] Wang C (2015). Biogeomorphic feedback between plant growth and flooding causes alternative stable states in an experimental floodplain. Adv Water Resour..

[3] Breidenbach B, Blaser MB, Klose M, Conrad R (2015). Crop rotation of flooded rice with upland maize impacts the resident and active methanogenic microbial community. Environ Microbiol..

[4] Mohammadi A, Costelloe JF, Ryu D (2017). Application of time series of remotely sensed normalized difference water, vegetation and moisture indices in characterizing flood dynamics of large-scale arid zone floodplains. Remote Sens Environ..

[5] Colmer TD, Voesenek LACJ (2009). Flooding tolerance: suites of plant traits in variable environments. Funct Plant Biol..

[6] Loreti E, Veen HV, Perata P (2016). Plant responses to flooding stress. Curr Opin Plant Biol..

[7] Winkel A (2016). Leaf gas films, underwater photosynthesis and plant species distributions in a flood gradient. Plant Cell Environ..

[8] Pucciariello C, Voesenek LACJ, Perata P (2014). Plant responses to flooding. Front Plant Sci.

[9] Benschop JJ (2005). Contrasting interactions between ethylene and abscisic acid in Rumex species differing in submergence tolerance. Plant J..

[10] Pucciariello C, Perata P (2013). Quiescence in rice submergence tolerance: an evolutionary hypothesis. Trends Plant Sci..

[11] Lei ST, Zeng B, Yuan Z, Xu SJ (2014). Changes in carbohydrate content and membrane stability of two ecotypes of Calamagrostis arundinacea growing at different elevations in the drawdown zone of the Three Gorges Reservoir. PloS One.

[12] Su XL, Zeng B, Huang WJ, Xu SJ, Lei ST (2012). Effects of the Three Gorges Dam on preupland and preriparian drawdown zones vegetation in the upper watershed of the Yangtze River, P.R. China. Ecol Eng..

[13] Su XL (2016). How does long-term complete submergence influence sex ratio and resource allocation of a dioecious shrub, *Salix variegata* Franch.?. Ecol Eng..

[14] Lin F, Liu JH, Zeng B, Pan XJ, Su XL (2016). Submergence Tolerance and Germination Dynamics of Roegneria nutans Seeds in Water-Level Fluctuation Zones with Different Water Rhythms in the Three Gorges Reservoir. Plos One.

[15] Li Y (2008). The effects of flooding on survival and recovery growth of the riparian plant Salix variegata Franch in Three Gorges reservoir region. Acta Ecol Sin..

[16] Lei ST, Zeng B, Xu SJ, Su XL (2012). Membrane responses of Salix variegata and Cinnamomum camphora to complete submergence in the Three Gorges Reservoir Region. Acta Ecol Sin..

[17] Bazihizina N (2016). Awaiting better times: A quiescence response and adventitious root primordia formation prolong survival under cadmium stress in Tetradenia riparia (Hochst.) Codd. Environ Exp Bot..

[18] Rønning B, Broggi J, Bech C (2016). Is basal metabolic rate associated with recruit production and survival in free‐living house sparrows?. Funct Ecol..

[19] Wone BWM, Madsen P, Donovan ER (2015). A strong response to selection on mass-independent maximal metabolic rate without a correlated response in basal metabolic rate. Heredity.

[20] Sadowska ET, Stawski C, Rudolf A (2016). Evolution of basal metabolic rate in bank voles from a multidirectional selection experiment. P. Roy Soc Lond B-Bio Sci..

[21] Brzęk P, Książek A, Ołdakowski A (2014). High basal metabolic rate does not elevate oxidative stress during reproduction in laboratory mice. J Exp Biol..

[22] Bailey-Serres J, Voesenek LACJ (2008). Flooding Stress: Acclimations and Genetic Diversity. Annu Rev Plant Biol..

[23] Gautam P, Nayak AK, Lal B (2014). Submergence tolerance in relation to application time of nitrogen and phosphorus in rice (Oryza sativa L.). Environ Exp Bot..

[24] Manzur ME, Grimoldi AA, Insausti P, Striker GG (2009). Escape from water or remain quiescent? Lotus tenuis changes its strategy depending on depth of submergence. Ann Bot..

[25] Kumagai, M. & Yahagi, N. Basal metabolic rate. In *Encyclopedia of Behavioral Medicine* (Gellman, M. D. & Turner, J. R.), 176–177 (2013).

[26] Waring, E. F. & Maricle, B. R. Photosynthetic variation and carbon isotope discrimination in invasive wetland grasses in response to flooding. *Environ Exp Bot*. 77–86 (2012).

[27] Müller M (2016). Decreased capacity for sodium export out of Arabidopsis chloroplasts impairs salt tolerance, photosynthesis and plant performance. Plant J..

[28] Sun X (2016). AsHSP17, a creeping bentgrass small heat shock protein modulates plant photosynthesis and ABA-dependent and independent signalling to attenuate plant response to abiotic stress. Plant Cell Environ..

[29] Voesenek LACJ, Bailey-Serres J (2015). Flood adaptive traits and processes: an overview. New Phytol..

[30] Londoño GA, Chappell MA, Castañeda MDR, Jankowski JE, Robinson SK (2016). Basal metabolism in tropical birds: latitude, altitude, and the ‘pace of life’. Funct Ecol..

[31] Choi JW, Pai SH (2004). Brief communication: Respiratory function is closely associated with basal metabolic rate in elderly persons. Ann Clin Lab Sci..

[32] Slot M, Kitajima K (2015). Whole-plant respiration and its temperature sensitivity during progressive carbon starvation. Funct Plant Biol..

[33] Monaghan RM (2015). A nuclear role for the respiratory enzyme CLK-1/COQ7 in regulating mitochondrial stress responses and longevity. Nature Cell Biol..

[34] O’Leary, B. M. & Plaxton, W. C. Plant respiration. In *eLS*, 1–11 (2016).

[35] Shingaki-Wells R, Millar AH, Whelan J, Narsai R (2014). What happens to plant mitochondria under low oxygen? An omics review of the responses to low oxygen and reoxygenation. Plant Cell Environ..

[36] Pan, R. C. Plant respiration. In *Plant Physiology Vol*. *4* (Pan, R. C, *et al*.*)*, 117–118 (2003).

[37] Voesenek LACJ (2003). De-submergence-induced ethylene production in *Rumex palustris*: regulation and ecophysiological significance. Plant J..

[38] Duca, M. Plant respiration. In *Plant Physiology*, 123–148 (2015).

[39] Luo FL, Zeng B, Chen T, Ye XQ (2007). Response to simulated flooding of photosynthesis and growth of riparian plant Salix variegata in the Three Gorges Reservoir region of China. J Plant Ecol (Chin Ver.)..

[40] Parad, G. A., Kouchaksaraei, M. T., Striker, G. G., Sadati, S. E., & Nourmohammadi, K. Growth, morphology and gas exchange responses of two-year-old seedlings to flooding stress. *Scand J Forest Res*. **31**(5), 10.1080/02827581.2015.1072240 (2016).

[41] Ye XQ, Zeng B (2013). Survival and carbohydrate storage in two tolerant plant species exposed to prolonged flooding in the Three Gorges Reservoir Region. Acta Hydrobiol Sin..

[42] Zhang, Y. H. Effects of flooding on growth and carbohydrate storage of *Salix variegata*. Master degree thesis, Southwest University: Chongqing (2006).

[43] Parad GA (2013). Some physiological and morphological responses of *Pyrus boissieriana* to flooding. Trees.

[44] Jackson MB (2008). Ethylene-promoted Elongation: an adaptation to submergence stress. Ann Bot..

[45] Phukan UJ, Mishra S, Shukla RK (2016). Waterlogging and submergence stress: affects and acclimation. Crit Rev Biotechnol..

[46] Parolin P (2009). Submerged in darkness: adaptations to prolonged submergence by woody species of the Amazonian floodplains. Ann Bot..

[47] Ayi, Q. L. *et al*. Oxygen absorption by adventitious roots promotes the survival of completely submerged terrestrial plants. *Ann Bot*. **118**(4), (2016). 10.1093/aob/mcw051 PMC5055620 27063366

[48] Jackson MB, Ismail AM (2015). Introduction to the Special Issue: Electrons, water and rice fields: plant response and adaptation to flooding and submergence stress. AoB Plant.

[49] Takahashi, H., Yamauchi, T., Colmer, T. D. & Nakazono, M. Aerenchyma formation in plants. In *Low-Oxygen Stress in Plants* (Dongen, J. T. van & Licausi, F.), 247–265 (2014).

[50] Xu JP (2014). Effects of light and dissolved oxygen on the phenotypic plasticity of Alternanthera philoxeroides in submergence conditions. Acta Ecol Sin..

[51] Li S, Reza PS, Jr DSF (2006). Partial flooding enhances aeration in adventitious roots of black willow (Salix nigra) cuttings. J Plant Physiol..

[52] Kim EJ (2014). Study on flooding tolerance of *Salix* Species for ecological restoration of the river. J Wetland Res..

